# Passive Smoking at Home by Socioeconomic Factors in a Japanese Population: NIPPON DATA2010

**DOI:** 10.2188/jea.JE20170243

**Published:** 2018-03-05

**Authors:** Minh Nguyen, Nobuo Nishi, Aya Kadota, Nagako Okuda, Hisatomi Arima, Akira Fujiyoshi, Yasutaka Nakano, Takayoshi Ohkubo, Hirotsugu Ueshima, Akira Okayama, Katsuyuki Miura

**Affiliations:** 1 Center for Epidemiologic Research in Asia, Shiga University of Medical Science, Shiga, Japan; 2 Division of Respiratory Medicine, Department of Internal Medicine, Shiga University of Medical Science, Shiga, Japan; 3 International Center for Nutrition and Information, National Institute of Health and Nutrition, National Institutes of Biomedical Innovation, Health and Nutrition, Tokyo, Japan; 4 Department of Public Health, Shiga University of Medical Science, Shiga, Japan; 5 Department of Health and Nutrition, University of Human Arts and Sciences, Saitama, Japan; 6 Department of Preventive Medicine and Public Health, Faculty of Medicine, Fukuoka University, Fukuoka, Japan; 7 Department of Hygiene and Public Health, Teikyo University School of Medicine, Tokyo, Japan; 8 Research Institute of Strategy for Prevention, Tokyo, Japan

**Keywords:** passive smoking, socioeconomic factors, national surveys

## Abstract

**Background:**

Long-term passive exposure to cigarette smoke has been reported to affect the health of non-smokers. This study aims to investigate the relationships among socioeconomic factors and passive smoking at home in the non-current smokers of a representative sample from a general Japanese population.

**Methods:**

Data are from NIPPON DATA2010. Among 2,891 participants, 2,288 non-current smokers (1,763 never smokers and 525 past smokers) were analyzed in the present study. Cross-sectional analyses were performed on the relationships among socioeconomic factors and passive smoking at home (several times a week or more) in men and women separately. Socioeconomic factors were employment, length of education, marital status, and equivalent household expenditure. Odds ratios (ORs) and 95% confidence intervals (CIs) were calculated using a multivariable logistic regression model.

**Results:**

The multivariable-adjusted model showed that employed women had a higher risk of passive smoking than unemployed women (OR 1.44; 95% CI, 1.06–1.96). Women with 9 years or less of education had a higher risk of passive smoking at home than women with 13 years and more of education (OR 2.37; 95% CI, 1.49–3.78). Single women had a lower risk of passive smoking at home (OR 0.53; 95% CI, 0.37–0.77) than married women. No significant associations were observed in men.

**Conclusions:**

An employed status, lower education, and being single were associated with passive smoking at home in the non-current smoking women of a representative Japanese population.

## INTRODUCTION

Long-term passive exposure to cigarette smoking has been reported to affect the health of non-smokers.^1^ Passive smoking involves the inhalation of the smoke of cigarettes, cigars, or pipes from others, and its existence has become a major health issue.^2^ Approximately 600,000 deaths worldwide were estimated to be attributed to second-hand smoke; 47% of deaths occurred in women, 28% in children, and 26% in men.^3^ Cardiovascular and respiratory diseases are adverse health outcomes among adults. Insufficient birth weight, sudden death, and middle ear infections were identified as consequences of second-hand smoke exposure among children.^4^ In order to improve the health of specific populations, a number of policies have been implemented.^5^^–^^8^ In 2008, the World Health Organization reported six evidence-based tobacco control policies that were the most effective at reducing exposure to cigarette and second-hand smoke.^5^

Relationships have been identified among passive smoking and sociodemographic factors. Exposure to second-hand smoke is high in homes, at workplaces, and in other public areas in low- and middle-income countries.^9^ Second-hand smoke exposure, which more frequently occurs at homes and in indoor workplaces, was shown to be higher among populations with a low socioeconomic status.^4^ A clearer understanding of the socioeconomic imbalance in passive smoking exposure may be useful for identifying possibilities to diminish health inequalities. Previous findings demonstrated that in low- and middle-income countries, as well as in high-income countries, approaches for the control of cigarette smoke, including smoke-free policies, are needed in order to change social standards by advancing smoking as unacceptable conduct, thereby protecting non-smokers from second-hand smoke exposure.^10^^,^^11^ However, limited nationally representative data are currently available on the relationships among passive smoking and socioeconomic factors in non-smokers in Japan.

This study aimed to investigate relationships among socioeconomic factors and passive smoking at home in non-current smokers in a representative sample from the general Japanese population that participated in the National Health and Nutrition Survey.

## METHODS

### Study population

In 2010, a prospective cohort study on cardiovascular diseases, the National Integrated Project for Prospective Observation of Non-communicable Disease and its Trends in the Aged 2010 (NIPPON DATA2010), was initiated.^12^ The study was implemented in addition to the National Health and Nutrition Survey of Japan (NHNS) in November 2010 (NHNS2010) and the Comprehensive Survey of Living Conditions (CSLC) in June 2010 (CSLC2010), which were conducted by the Ministry of Health, Labor and Welfare of Japan. The details of NHNS2010 and CLSLC2010 have already been reported.^13^^–^^15^

In November 2010, 8,815 residents aged 1 year and older from 300 randomly selected districts throughout Japan participated in the dietary survey for NHNS2010. Among the 3,873 participants of NHNS2010 aged 20 years or older with blood tests, 2,891 (1,236 men and 1,655 women) agreed to participate in the baseline survey of NIPPON DATA2010. The baseline survey for NIPPON DATA2010 included an electrocardiogram, urinalysis, and questionnaire on cardiovascular diseases, since those were not part of NHNS2010.^12^ Informed consent was collected before study participants were enrolled.^16^

**Figure 1.**
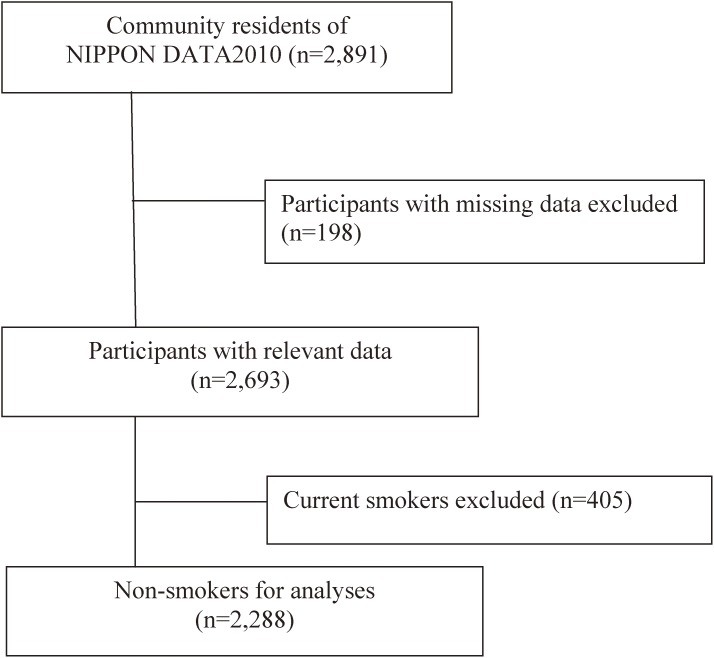
Study participants

Of the 2,891 participants, 198 were excluded because of missing data (length of education, smoking status, passive smoking, marital status, employment status, and household expenditure). Among the remaining 2,693 participants, 405 current smokers were excluded. Thus, 2,288 participants (834 men and 1,454 women; 1,763 never smokers and 525 past smokers) were analyzed in the present study (Figure 1). The Institutional Review Board of Shiga University of Medical Science (No. 22–29, 2010) approved this study.

### Socioeconomic status

Information on socioeconomic factors was collected from self-administered questionnaires for NHNS2010 (employment status), CSLC2010 (monthly household expenditure of 2010 May, the month before CSLC2010), and NIPPON DATA2010 (length of education, marital status [married or unmarried], and living status [living alone or not living alone]). Socioeconomic factors were classified according to (1) employment status (employed [including self-employed] or unemployed), (2) length of education (9 years or less, 10–12 years, or 13 years or longer), (3) marital status (married, single [including never married, divorced, and widowed]), (4) Equivalent household expenditure (EHE) (10–99 thousand Japanese Yen/month or 100–4,000 thousand Japanese Yen/month). EHE was calculated as household expenditure divided by the square root of the number of family members.

In the questionnaire for NHNS2010, participants were asked “*Have you inhaled the cigarette smoke of someone else (passive smoking) in the last month?*” and prompted to give its frequency (as either “almost every day”, “several times a week”, “once a week”, “once a month”, or “not at all”) for each location provided in the questionnaire (“at home”, “at the workplace”, “at school”, “at a restaurant”, “at a game hall”, and “other places”). We defined passive smoking at home as passive smoking at home several times a week or more (“almost every day” or “several times a week”).

### Statistical analysis

The characteristics of the study participants are presented as a number and percentage for categorical variables. The relationships among passive smoking at home and socioeconomic factors were estimated using a logistic regression model. Model 1 was adjusted for age and past smoking (additionally adjusted for house ownership only in EHE). Model 2 included all variables (4 socioeconomic factors) simultaneously adjusted for each other, in addition to age, past smoking, and house ownership. Analyses were performed on men and women separately. The results of logistic regression analyses were reported as odds ratios (ORs) with corresponding 95% confidence intervals (CIs). A *P* value <0.05 was considered to be significant. All analyses were performed using the statistical software SPSS for windows version 23.0 (IBM Corp., Armonk, NY, USA).

## RESULTS

### Participant characteristics

The characteristics of participants are shown in Table 1. Among 2,288 participants, men and women in the older age group (≥60 years) accounted for 66.1% and 55.4%, respectively. Employed men accounted for 56.7% of the sample, and employed women comprised 41.9%. The percentages of men and women with an education of 9 years or less were 24.3% and 23.6%, respectively. Married men and women accounted for 82.5% and 73.2% of the sample, respectively. Men and women with EHE per month between 10 and 99 thousand Japanese Yen accounted for 24.2% and 24.3% of the sample, respectively. Passive smoking at home several times a week or more was reported by 5.9% of men and 15.9% of women.

**Table 1.** Characteristics of participants by gender among non-smokers in NIPPON DATA2010 (*n* = 2,288)

	Men (*n* = 834) *n* (%)	Women (*n* = 1,454) *n* (%)
Age group		
Younger (20–39 years)	99 (11.9)	248 (17.1)
Middle age (40–59 years)	184 (22.1)	400 (27.5)
Older (≥60 years)	551 (66.1)	806 (55.4)
Employment status		
Employed	473 (56.7)	609 (41.9)
Unemployed	361 (43.3)	845 (58.1)
Length of education		
≤9 years	203 (24.3)	343 (23.6)
10–12 years	341 (40.9)	663 (45.6)
≥13 years	290 (34.8)	448 (30.8)
Marital status		
Married	688 (82.5)	1,065 (73.2)
Single	146 (17.5)	389 (26.8)
Equivalent household expenditure per month		
10–99 thousand Japanese yen	202 (24.2)	353 (24.3)
100–4,000 thousand Japanese yen	632 (75.8)	1,101 (75.7)
Passive smoking at home		
Several times a week or more	49 (5.9)	231 (15.9)
Once a week or less	785 (94.1)	1,223 (84.1)

The characteristics of passive smoking at home by socioeconomic factors are presented in Table 2. Among men, significant differences were observed in the prevalence of passive smoking with age and employment status. The highest percentage of passive smoking was observed in the younger age group (13.1%). The prevalence of passive smoking was higher in employed men than in unemployed men.

**Table 2.** Characteristics of passive smoking at home by socioeconomic status and gender, NIPPON DATA2010 (*n* = 2,288)

	Men (*n* = 834)			Women (*n* = 1,454)		
	
	Several times a week or more *N* (%)	Once a week or less *N* (%)	*P* Value (for men)	Several times a week or more *N* (%)	Once a week or less *N* (%)	*P* Value (for women)
Age group						
Younger (20–39 years)	13 (13.1)	86 (86.9)	0.003	51 (20.6)	197 (79.4)	<0.001
Middle age (40–59 years)	12 (6.5)	172 (93.5)		80 (20.0)	320 (80.0)	
Older (≥60 years)	24 (4.4)	527 (95.6)		100 (12.4)	706 (87.6)	
Employment status						
Employed	35 (7.4)	438 (92.6)	0.032	119 (19.5)	490 (80.5)	0.001
Unemployed	14 (3.9)	347 (96.1)		112 (13.3)	733 (86.7)	
Length of education						
≤9 years	12 (5.9)	191 (94.1)	0.594	59 (17.2)	284 (82.8)	0.729
10–12 years	23 (6.7)	318 (93.3)		104 (15.7)	559 (84.3)	
≥13 years	14 (4.8)	276 (95.2)		68 (15.2)	380 (84.8)	
Marital status						
Married	39 (5.7)	649 (94.3)	0.582	190 (17.8)	875 (82.2)	0.001
Single	10 (6.8)	136 (93.2)		41 (10.5)	348 (89.5)	
Equivalent household expenditure per month						
10–99 thousand JPY	16 (7.9)	186 (92.1)	0.156	54 (15.3)	299 (84.7)	0.728
100–4,000 thousand JPY	33 (5.2)	599 (94.8)		177 (16.1)	924 (83.9)	

JPY, Japanese yen.

A bivariate comparison was made with the chi-squared test.

In women, significant differences were observed in the prevalence of passive smoking by age, employment status, and marital status (Table 2). The highest prevalence of passive smoking at home was observed in the younger age group (20.6%). The prevalence of passive smoking was higher in employed women (19.5%) and married women (17.8%) compared with that in other women.

### Relationships among socioeconomic factors and passive smoking at home

Relationships among socioeconomic factors and passive smoking at home are shown in Table 3. An age and past smoking-adjusted model (model 1) showed no significance in the relationships among passive smoking at home and employment status, length of education, marital status, and EHE per month in men. Women with 9 years or less of education had a higher risk of passive smoking at home than women with 13 years or more of education (OR 2.06; 95% CI, 1.31–3.25). Single women had a lower risk of passive smoking at home (OR 0.57; 95% CI, 0.40–0.82) than married women. No significant relationships were observed between passive smoking at home and employment status or EHE per month in women.

**Table 3.** Odds ratios (95% confidence intervals) of passive smoking at home for socioeconomic status by sex, NIPPON DATA2010 (*n* = 2,288)

	Men (*n* = 834)		Women (*n* = 1,454)	
	
	Model 1^a^	Model 2^b^	Model 1^a^	Model 2^b^
Employment status				
Employed	1.26 (0.60–2.65)	1.29 (0.60–2.81)	1.35 (0.99–1.83)	**1.44 (1.06–1.96)**
Unemployed	1.00	1.00	1.00	1.00

Length of education				
≤9 years	2.42 (0.99–5.90)	2.26 (0.90–5.67)	**2.06 (1.31–3.25)**	**2.37 (1.49–3.78)**
10–12 years	1.75 (0.87–3.53)	1.73 (0.86–3.51)	1.36 (0.95–1.94)	1.37 (0.96–1.95)
≥13 years	1.00	1.00	1.00	1.00

Marital status				
Single	0.84 (0.38–1.84)	0.74 (0.33–1.69)	**0.57 (0.40–0.82)**	**0.53 (0.37–0.77)**
Married	1.00	1.00	1.00	1.00

Equivalent household expenditure per month				
10–90 thousand JPY	0.67 (0.36–1.25)	0.71 (0.37–1.35)	1.04 (0.74–1.45)	1.15 (0.81–1.61)
100–4,000 thousand JPY	1.00	1.00	1.00	1.00

JPY, Japanese yen.

^a^Model 1 is adjusted for age and past smoking (additionally adjusted for house ownership for equivalent household expenditure only).

^b^Model 2 includes all variables simultaneously adjusting for each other, in addition to age, past smoking, and house ownership.

A multivariable-adjusted model (model 2) showed that employed women had a higher risk of passive smoking than unemployed women (OR 1.44; 95% CI, 1.06–1.96). Women with 9 years or less of education had a higher risk of passive smoking at home than women with 13 years or more of education (OR 2.37; 95% CI, 1.49–3.78). Single women had a lower risk of passive smoking at home (OR 0.53; 95% CI, 0.37–0.77) than married women. There was no significant relationship among passive smoking at home and employment status, length of education, marital status, or EHE per month in men.

## DISCUSSION

Our results showed that an employed status, low education, and being single were associated with passive smoking at home in the non-current smoking women of a representative Japanese population. No relationships were observed among passive smoking at home and employment status, length of education, marital status, or EHE per month in men.

Limited information is available on the status of passive smoking in non-smokers in Japan. Thus, findings from the associated factors of passive smoking may be beneficial for reducing the prevalence of passive smoking and protecting those at high risk. Our study was one of the largest population-based surveys to investigate the relationships among passive smoking at home and socioeconomic factors in Japan.

In the present study, an association was observed between employment status and passive smoking at home in women. This may be explained by employed women being exposed to second-hand smoke from other household members. Our results are consistent with the findings of a study in the United States. Employed women with manual working occupations and lower educational fulfilment were more likely to be exposed to second-hand smoke at home.^17^ This study also showed that employed women were not living alone.^17^ Thus, our results suggest that the smoking status of the family unit will be a solid indicator of receiving a smoke-free-at-home approach, trailed by smoking status of the individual. In addition, employment status in the present study included the self-employed; a significant number of self-employed women reported passive smoking at home.

Lower education (9 years or less) in women was associated with a higher risk of passive smoking at home than that of 13 years or more. Possible explanations for these results are that less educated populations may lack information and knowledge on passive smoking. Furthermore, the husbands of women with lower education may also be less educated and have a higher smoking rate (data not shown). A cross-sectional study by the 2005 Korea National Health and Nutrition Examination Survey assessed exposure to environmental tobacco smoke among South Korean adults and showed that the odds of an elementary and middle education in non-smokers were 1.52 (95% CI, 1.23–1.87) and 1.88 (95% CI, 1.47–2.40), respectively, relative to college and higher.^18^ In a study from New Zealand, environmental tobacco smoke exposure was minimal among participants with a university education (30%, mean exposure 16 minutes per week, consistently exposed to some environmental tobacco smoke).^19^ Previous studies from high-income countries reported similar findings.^20^^,^^21^ In the present study, although the estimated ORs in men were high for an education of 9 years or less and 10–12 years (2.27 and 1.74, respectively), statistical power was insufficient.

A relationship was not observed between marital status and passive smoking at home in men. However, single women showed a lower risk of passive smoking at home. This may be explained by Japanese women spending more time at home, and men being more likely to be a current smoker than women. Thus, married women may be exposed to more passive smoking opportunities from their husbands in the home. The higher prevalence of passive smoking in married women suggests an urgent need to control tobacco for non-smokers at home, including their children.

To the best of our knowledge, this is the first study in Japan to examine EHE with passive smoking at home. However, no significant associations were detected. We did not use income to evaluate economic status because older retired people with a higher socioeconomic status have a lower income but higher expenditure. In the present study, a higher education was related to a lower risk of passive smoking, whereas higher EHE was not. This may reflect a weaker relationship between education and economic status in Japan.

Our study is unique in several ways. Unlike previous studies, we indicated associated factors for exposure to passive smoking based on location, gender, and the frequency of exposure. In this study, our analyses concentrated on passive smoking at home because this is one of the two most common settings (at home and at the workplace) of second-hand smoke exposure.^4^ Another strength of the present study is the large population-based sample of a national survey recruited across Japan, which enforces the generalizability of our results to the Japanese population.

The limitations of the present study include the properties of the cross-sectional study and the recall bias of all self-reported questionnaire interviews. The reporting bias of socioeconomic status (ie, under-reporting of a very high status or over-reporting of a very low status) may have weakened the relationships among socioeconomic factors and passive smoking at home. We depended on self-reported measures of home exposure to second-hand smoke without biological markers (eg, cotinine levels). Recent evidence indicated that without such biomarkers, the true prevalence of second-hand smoke exposure is underestimated.^22^^,^^23^ In addition, since this study was cross-sectional in nature, we were unable to assess whether there was a causal relationship; however, reverse causation was not likely to occur. Furthermore, the results of the present analysis may not be generalized to other countries in which lifestyles, access to medical care, and medical insurance coverage are different from Japan.

The results of the present study indicate that socioeconomic inequalities exist in exposure to passive smoking at home. This evidence provides good support for comprehensive smoke-free policies. The expansion of the quantity of nations with extensive tobacco control policies demonstrates that viable laws are moderately simple to pass and uphold and include almost no cost.^24^ For example, recent evidence showed that non-smoke regulations in England were related to a decrease in the confirmation of respiratory tract contamination in lower-socioeconomic-status children.^25^ A few studies indicated that open spots and home smoking bans should not be separated. Shielding individuals from the dangers of second-hand smoke additionally decreases the probability that children will begin smoking.^26^^–^^29^ In order to lessen imbalances in smoking, the usage of tobacco control regulations needs to be reinforced, particularly intercessions that appear to be of genuine value, such as expanding tobacco taxes.^30^ Centered endeavors are required to address social standards exposing others to second-hand smoke (eg, mindfulness through broad communication crusades and other instructive mediations), with a focus on financially hindered gatherings. Smoke-free approaches were previously demonstrated to have an impact on social standards regarding exposure to second-hand smoke at home.^10^

In conclusion, in this representative sample of the Japanese population, we found that an employed status, being single, and lower education were positively associated with passive smoking exposure at home in women.
